# FITNESS Acts as a Negative Regulator of Immunity and Influences the Plant Reproductive Output After *Pseudomonas syringae* Infection

**DOI:** 10.3389/fpls.2021.606791

**Published:** 2021-02-04

**Authors:** Diego Alberto Mengarelli, Lara Roldán Tewes, Salma Balazadeh, María Inés Zanor

**Affiliations:** ^1^Instituto de Biología Molecular y Celular de Rosario (IBR-CONICET) Ocampo y Esmeralda PREDIO CCT-Facultad de Ciencias Bioquímicas y Farmacéuticas (UNR), Rosario, Argentina; ^2^Max Planck Institute of Molecular Plant Physiology, Potsdam, Germany

**Keywords:** pathogen, Arabidopsis, salicylic acid, NPR1, defense

## Abstract

Plants, as sessile organisms, are continuously threatened by multiple factors and therefore their profitable production depends on how they can defend themselves. We have previously reported on the characterization of *fitness* mutants which are more tolerant to environmental stresses due to the activation of defense mechanisms. Here, we demonstrate that in *fitness* mutants, which accumulate moderate levels of salicylic acid (SA) and have SA signaling activated, pathogen infection is restricted. Also, we demonstrate that NPR1 is essential in *fitness* mutants for SA storage and defense activation but not for SA synthesis after *Pseudomonas syringae* (*Pst*) infection. Additionally, these mutants do not appear to be metabolically impared, resulting in a higher seed set even after pathogen attack. The FITNESS transcriptional network includes defense-related transcription factors (TFs) such as *ANAC072*, *ORA59*, and *ERF1* as well as jasmonic acid (JA) related genes including *LIPOXYGENASE2 (LOX2), CORONATINE INSENSITIVE1 (COI1)*, *JASMONATE ZIM-domain3* (*JAZ3*) and *JAZ10*. Induction of *FITNESS* expression leads to *COI1* downregulation, and to *JAZ3* and *JAZ10* upregulation. As COI1 is an essential component of the bioactive JA perception apparatus and is required for most JA-signaling processes, elevated *FITNESS* expression leads to modulated JA-related responses. Taken together, FITNESS plays a crucial role during pathogen attack and allows a cost-efficient way to prevent undesirable developmental effects.

## Introduction

Annual crop yield losses due to pathogen attack are a major concern for producers. Although chemical control has been increased, the use of synthetic chemicals represents a threat to global food security and agricultural sustainability (Nelson et al., 2017), and many farmers have started to look into more natural ways for food production. Enhancing plant resistance is an alternative way to avoid the use of chemicals. However, genes that play roles in disease resistance might also affect other important traits such as the response to abiotic factors or yield.

Some plant transcription factors (TFs) have been described as master regulators of essential aspects – such as development and stress response – using different approaches. We recently described the function of *FITNESS* in *Arabidopsis thaliana*, which leads to the deregulation of reactive oxygen species (ROS) levels *in planta* when *FITNESS* is overexpressed using the CaMV *35S* promoter (Osella et al., 2018). FITNESS protein possesses a single CCT (CONSTANS, CONSTANS-like, and TOC1) domain, first described in the protein CONSTANS (Robson et al., 2001). It is included in a family of uncharacterized genes named CCT motif family genes (Cockram et al., 2012). Genes encoding CCT domain proteins have been implicated in processes such as photoperiodic flowering (Putterill et al., 1995), regulation of the circadian rhythm (Strayer et al., 2000), and plant architecture (Ordoñez-Herrera et al., 2018). There are also some examples of CCT members involved in biotic stresses, like ZmCCT10, which is responsive to Gibberella stalk rot resistance in maize (*Zea mays*; Wang et al., 2017), and OsCOL9, identified as an early response gene in rice (*Oryza sativa*) after *Magnaporthe oryzae* infection (Liu et al., 2016).

Under normal growth conditions, transgenic Arabidopsis plants constitutively overexpressing *FITNESS* (*FITNESS*_ox_ lines) accumulate high ROS levels and show reduced growth and seed set. On the contrary, plant performance was increased in the mutant *fitness-1* and *fitness-2*, compared to Col-0 wild type (WT), resulting in a higher seed set. To better understand the observed phenotypes’ basis, we previously analyzed the global transcriptome and metabolome of the lines with altered *FITNESS* expression. Transcript abundance of genes related to the biosynthesis and signaling of the plant hormone salicylic acid (SA) was increased in *fitness* mutants. In line with this, a moderate increase in the levels of free SA was measured (Osella et al., 2018).

Salicylic acid plays an essential role in plant defense against biotrophic and hemibiotrophic pathogens (Fu and Dong, 2013). In Arabidopsis, the bacterium *Pseudomonas syringae* causes extensive chlorosis and necrotic spots in leaves (Whalen et al., 1991), and basal resistance is predominantly dependent on SA (Wildermouth et al., 2001). After the pathway is activated at the infection site, a similar response is triggered in distal parts of the plant, inducing a broad-spectrum resistance called systemic acquired resistance or SAR (Conrad et al., 2015). The regulatory protein NON-EXPRESSOR OF PATHOGENESIS-RELATED GENES 1 (NPR1), which acts downstream of SA, plays an important role in SA-dependent defense signaling (Backer et al., 2019). Mutations in this gene impair the transcriptional reprograming exerted by SA and the establishment of SAR. NPR1 activation depends on the redox state of the cell. SA accumulation leads to a reduced cellular environment, which triggers the reduction of an oligomeric cytosolic NPR1 complex and its translocation into the nucleus. Once there, it interacts with basic leucine-zipper TFs, such as the TGACG-binding TFs (TGAs), and activates the expression of defense-related genes called *PATHOGENESIS-RELATED* (*PR*) genes. *PR1* is one of the best-characterized genes of the *PR* family, and experimentally it is used as a robust marker for SA-responsive gene expression. Expression of both *NPR1* and *PR1*, among others, is elevated in *fitness* mutants, indicating a constitutive activation of defense responses in the absence of pathogen stress. Constitutive activation of defense responses has frequently been associated with plant growth and productivity penalties. The general assumption is that resources are channeled toward defense responses, thereby compromising plant growth (Zhu et al., 2013). For example, the Arabidopsis mutant *suppressor of npr1*, *constitutive1* (*snc1*), which accumulates SA and shows NPR1-independent pathogen resistance, is dwarf due to a constitutive defense response (Zhang et al., 2003). Also, the overexpression of Arabidopsis *NPR1* (*AtNPR1*) in rice leads to broad-spectrum resistance associated with the development of a lesion mimic/cell death phenotype and decreased seed production (Fitzgerald et al., 2004; Quilis et al., 2008).

How defense responses repress plant growth at the molecular level is not well understood. Hormone crosstalk has emerged as a major player in regulating tradeoffs needed to balance growth and defense (Hout et al., 2014). Ample evidence exists that SA- and jasmonic acid (JA)-dependent signal transduction pathways cross-communicate during plant defense and enable plants to mount responses specifically tailored to the inducing attacker, improving defense responses. The interplay between SA- and JA-responses boosts the immune response against single attackers (Spoel and Dong, 2008). By analyzing plants with altered *FITNESS* expression levels, we aimed to determine whether SA pathway activation exerted in *fitness* mutants triggers an effective defense against bacterial attack and whether this response is associated with a decline in seed productivity.

The present study demonstrates a strongly enhanced resistance to *Pseudomonas syringae* pv. *tomato* DC3000 (*Pst*) in *fitness* mutants. Given that the exact mechanism by which SA activates NPR1 is not completely understood yet (Backer et al., 2019), *fitness* mutants provide an important genetic model for investigating the basis for enhanced plant resistance induction. Additionally, a higher yield relative to WT was measured in *fitness* mutants after pathogen attack. Our results suggest that low levels of FITNESS lead to the optimization of the stress responses gene network to integrate dynamic environmental inputs. This reconfiguration acts synergistically to maximize the plant’s reproductive success.

## Materials and Methods

### General

Chemicals and reagents were obtained from Sigma-Aldrich (St Louis, MO, United States) or Merck (Buenos Aires, Argentina). Standard molecular techniques were performed as described (Sambrook and Russell, 2001). DNA sequencing was performed by the University of Maine DNA sequencing facility (United States, Orono, ME). For sequence analyses, the tools provided by the National Center for Biotechnology Information^1^, the Arabidopsis Information Resource (TAIR)^2^, WolF Sort^3^, and GPS-SUMO 2.0^4^ were used. Restriction enzymes for cloning and reagents for quantitative real-time PCR (qPCR) were provided by Promega and Invitrogen Life Technologies (Buenos Aires, Argentina).

### Constructs and Plants

*Arabidopsis thaliana* accession Col-0 was employed as wild type in all experiments. *FITNESS* loss-of-function mutants (*fitness-1* and *fitness-2*), *FITNESS* overexpressing line1 (*FITNESSox*_1_) and a mutant line rescued by constitutive overexpression of the full-length *FITNESS* cDNA transcriptionally fused to the cauliflower mosaic virus 35S promoter (*fitness-1*/*35S-FITNESS*) used in this work were previously described (Osella et al., 2018). The double knockout mutant deficient in both *NPR1* and *FITNESS* was generated by crossing the two single knockout mutants derived from Col-0 and self-pollination of the F1 generation. For selection of the *fitness-1*/*npr1* knockout line, genomic DNA leaf extracts of the F2 generation were prepared from 25-day-old plants according to Rezadoost et al. (2016). The mutation of the *FITNESS* allele was determined by PCR amplification using primers FITNESS_For, FITNESS_Rev, specific for the WT allele amplification and the pair promFITNESS_For and LB for the T-DNA insertion. The primer pair PP2a_For and PP2a__Rev was used as control of amplification. *npr1-1* single mutation was confirmed by sequencing using primers npr1_For and npr1_Rev. (Supplementary Table 1 and Supplementary Figure 1). Relative transcript levels of *FITNESS* in these lines are shown in Supplementary Figure 2.

For the establishment of an inducible overexpression (IOE) construct, the *FITNESS* CDS was amplified by PCR with primers that contain a *Pac*I or *Spe*I site (Supplementary Table 1). PCR products were ligated into pGEM-T Easy vector (Promega, Mannheim, Germany). After sequence confirmation, the vector was digested with *Pac*I and *Spe*I, and the fragment containing the *FITNESS* CDS was ligated into the pER8 vector (Zuo et al., 2000). Constructs were transformed into Arabidopsis Col-0 wild type using *Agrobacterium tumefaciens* (strain GV3101)-mediated transformation employing a floral dip method. Arabidopsis IOE lines were selected on MS medium containing hygromycin. Subsequently, stable transgenic T3 lines showing an increased expression level by qPCR after estradiol induction were used for detailed analysis. For expression experiments, total RNA was isolated from 2-week-old *FITNESS*-IOE plants after treatment with 10 μM estradiol in 0.01% Silwet 77 for 4 and 6 h; plants treated as before but without estradiol were used as controls.

### Plant Growth Conditions

Individual Arabidopsis plants were grown in controlled growth chambers in 6-cm pots at approximately 70% relative humidity with a 16 h light/8 h dark period for long day (LD) conditions (23°C, 120 μmol m^–2^ s^–1^). To perform all experiments, all plants were grown alongside each other under carefully controlled conditions. All experiments were repeated at least three times.

For root growth analysis, surface-sterilized seeds were germinated on 0.5× MS plates, supplemented with 0.8% agar. After 4 days, seedlings were transferred to new plates containing 0, 10, or 50 μM Methyl Jasmonate (Sigma-Aldrich) and placed vertically for 12 days in a controlled growth chamber under the same conditions as stated before.

### Bacterial Strain, Plant Inoculation Conditions, and Bacterial Proliferation Assay

The virulent hemibiotrophic bacterial pathogen *Pseudomonas syringae* pv. *tomato* DC3000 (*Pst*) was used for infections as described in Liu et al. (2015). Briefly, bacteria were suspended in sterile 10 mM MgCl_2_ at approximately 10^7^ c.f.u. ml^–1^. The suspension was infiltrated into fully expanded Arabidopsis leaves through the abaxial surface. Three leaves of six biological replicates per genotype were inoculated. Leaf discs were taken from the inoculated leaves 30 h post-inoculation. To assess bacterial growth each sample was plated at least three times. After incubation at 28°C for 48 h, bacterial colonies were counted.

### RNA Extraction and qPCR

Total RNA was extrated using TRIzol (Invitrogen Life Technologies) following the manufacturer’s procedure. Three biological replicates were prepared for each genotype. RNA quality and quantity, as well as RNA reverse transcription and qPCR, were performed as previously described (Osella et al., 2018). For qPCR, primer sequences are given in Supplementary Table 1. PCR reactions were carried out in a Mastercycler ep Realplex thermocycler (Eppendorf, Westbury, NY, United States) using a SYBR Green fluorescence-based assay. The relative expression ratio for each gene was calculated as previously described (Pfaffl, 2001). The PCR efficiency for each reaction was calculated based on the profile of the emitted fluorescence in the exponential phase (Rutledge and Stewart, 2008). Transcript levels were normalized to the transcript level of *PROTEIN PHOSPHATASE 2A* (*PP2A*, At1g13320) gene (Czechowski et al., 2005). Reagents for qPCR were provided by Promega and Invitrogen Life Technologies (Buenos Aires, Argentina).

### Quantification of Damaged Leaf Area

The relative damage area was measured using ImageJ software at 6 days after infection with a bacterial suspension of *Pst*. Measurements are relative to the total leaf area. At least nine leaves per genotype were used for this analysis.

### Determination of SA Levels

Free and conjugated SAs were extracted from leaves (200 mg fresh weight) of 3-week-plants using the procedure described by Aboul-Soud et al. (2004). Three biological replicates were prepared for each genotype. Gas chromatography-mass spectrometry (GC-MS) was used to measure free and conjugated SA, and their absolute concentrations (nmol g FW^–1^) were determined by comparison with calibration curve response ratios of various concentrations of standard solutions, including the internal standard ribitol (Roessner-Tunali et al., 2003; Zanor et al., 2009).

### Statistical Analyses

Statistical analysis were performed using Student’s *t-*test embedded in the Microsoft Excel software. Only a return of *P* < 0.05 was designated statistically significant. For multiple comparisons, one-way analysis of variance (ANOVA) followed by Fisher’s least significant difference multiple comparison test was used. Statistically significant differences (*P* < 0.05) are indicated by different letters. The multiple comparison tests as well as the correlation coefficient and significances between phytohormone and transcript levels were calculated using the InfoStat software (Di Rienzo et al., 2011).

## Results

### The Knock-Down of *FITNESS* Reveals Its Role in Pathogen Resistance Against *Pseudomonas syringae* pv. *tomato* DC3000

**FIGURE 1:**
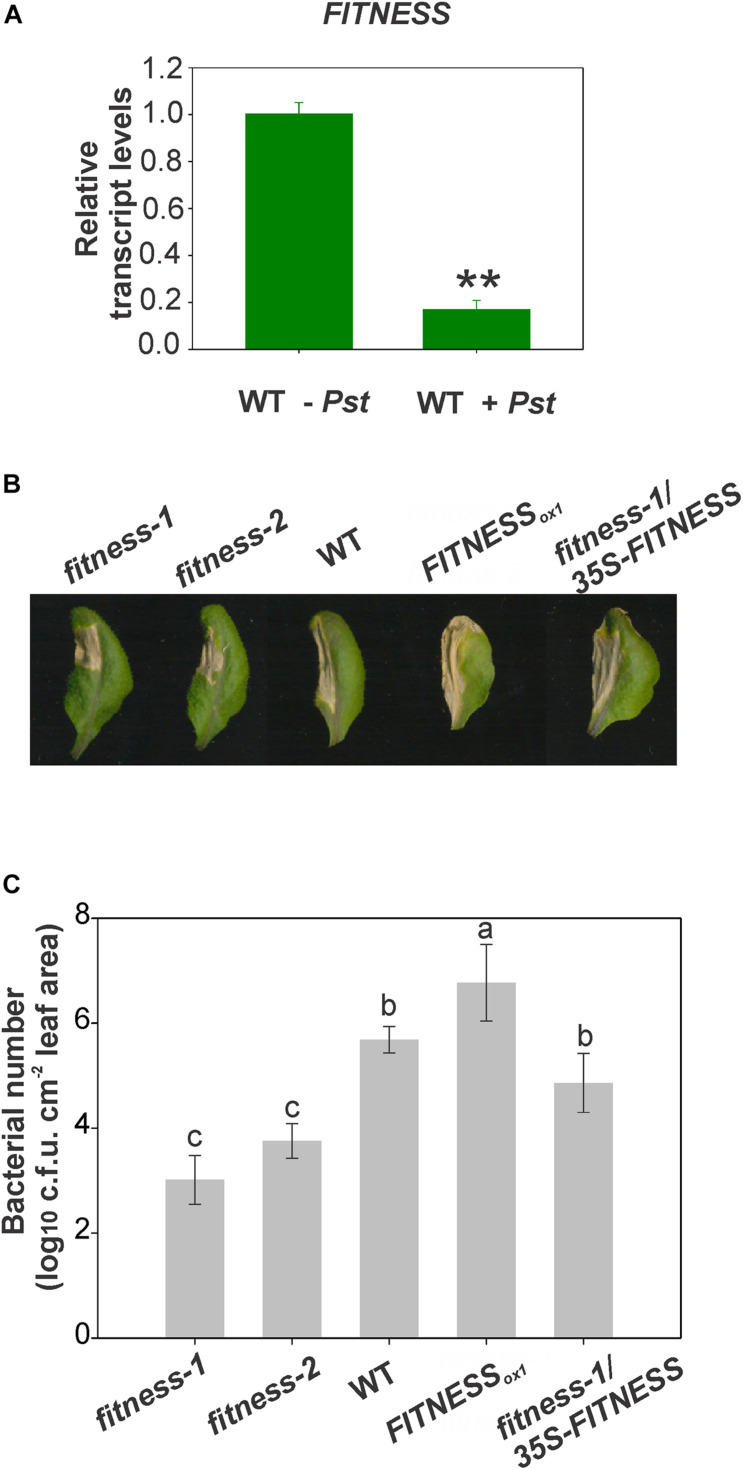
FITNESS knock down leads to *Pst* resistance. **(A)** *FITNESS* expression is reduced upon pathogen attack. RNA from 25-day-old wild-type *Arabidopsis thaliana* plants mock-infiltrated (WT-*Pst*) or after 24 h of *Pseudomonas syringae* pv *tomato* DC3000 (WT + *Pst*) treatment was used. Means of three biological replicates are shown; error bars represent SE. Statistical analyses were performed using Student’s *t*-test embedded in the Microsoft Excel software, statistically significant differences (*P* < 0.01) are indicated by **. **(B)** *FITNESS* is involved in disease resistance to *Pst* in Arabidopsis. Disease symptoms from representative leaves of 4-week-old plants expressing different levels of *FITNESS* are shown. Photographs were taken 4 days post-infection. **(C)** Bacterial titers, expressed as log10 (c.f.u. cm^–2^ leaf area), in the leaves of 4-week-old plants infiltrated with *Pst*. The experiments were repeated three times with similar results. Values were averaged from at least six biological replicates per genotype, and error bars represent SE. Bars with the same letter indicate no significant difference between the samples (analysis of variance + Fisher least significant difference, *P* < 0.05).

In a previous study, we presented evidence that the Arabidopsis *fitness* mutants exhibit increased tolerance to oxidative stress associated with an increase in plant productivity (Osella et al., 2018). The mutants also accumulated moderate SA levels accompanied by an induction of several SA-signaling marker genes, such as *NPR1*, *ENHANCED DISEASE SUSCEPTIBILITY1* (*EDS1*), and *PHYTOALEXIN DEFICIENT4* (*PAD4*). The induction of SA responses led us to test whether this activation of defenses is protective to the plant when challenged with pathogens. As mentioned before, SA plays an essential role in plant defense against biotrophic and hemibiotrophic pathogens. We, therefore, analyzed the expression of *FITNESS* upon *Pst* infection. Leaf material was used to test *FITNESS* transcripts levels after inoculation of Arabidopsis WT plants with the bacterial pathogen. After 24 h of infection, *FITNESS* transcript levels were significantly reduced (Figure 1A). To elucidate the possible involvement of *FITNESS* in plant defense against bacterial pathogens we tested Arabidopsis plants with altered *FITNESS* expression levels for their response to *Pst* infection, including the *fitness-1* and -*2* mutants (a T-DNA insertion mutant and a CRISPR/Cas9 mutant, respectively), and a transgenic line overexpressing *FITNESS* under the control of the *35S* CaMV promoter (hereafter, *FITNESS_ox__1_*), as previously described (Osella et al., 2018). Pressure-infiltration with *Pst* (1 × 10^7^ c.f.u. ml^–1^) was used to inoculate WT plants along with the mentioned lines. Compared with WT, *fitness-1* and -*2* exhibited enhanced disease resistance while the *FITNESS_ox__1_* line showed increased susceptibility toward *Pst*. Extended chlorosis was observed in all lines tested except the *fitness* mutants. Finally, the infection resulted in accelerated cell death in the inoculated leaves (Figure 1B). We also monitored the ability of *Pst* to multiply endophytically in the inoculated tissue and observed greater bacterial proliferation in *FITNESS_ox__1_* than WT plants (at 30 h post-inoculation; hpi). The opposite was evident in *fitness-1* and -*2* plants that had a lower bacterial titer than WT plants suggesting that the absence of FITNESS is associated with bacterial growth restriction (Figure 1C). To further explore these observations, we analyzed the *fitness-1*/*35S-FITNESS* line after pathogen attack. We observed a higher bacterial load in this line than in the *fitness-1* mutant, similar to that observed in the WT (Figure 1C). Taken together, our data suggest that FITNESS acts as a negative regulator of immunity to a bacterial pathogen.

### Salicylic Acid-Triggered Defense in *Fitness* Mutants Is NPR1-Dependent

**FIGURE 2:**
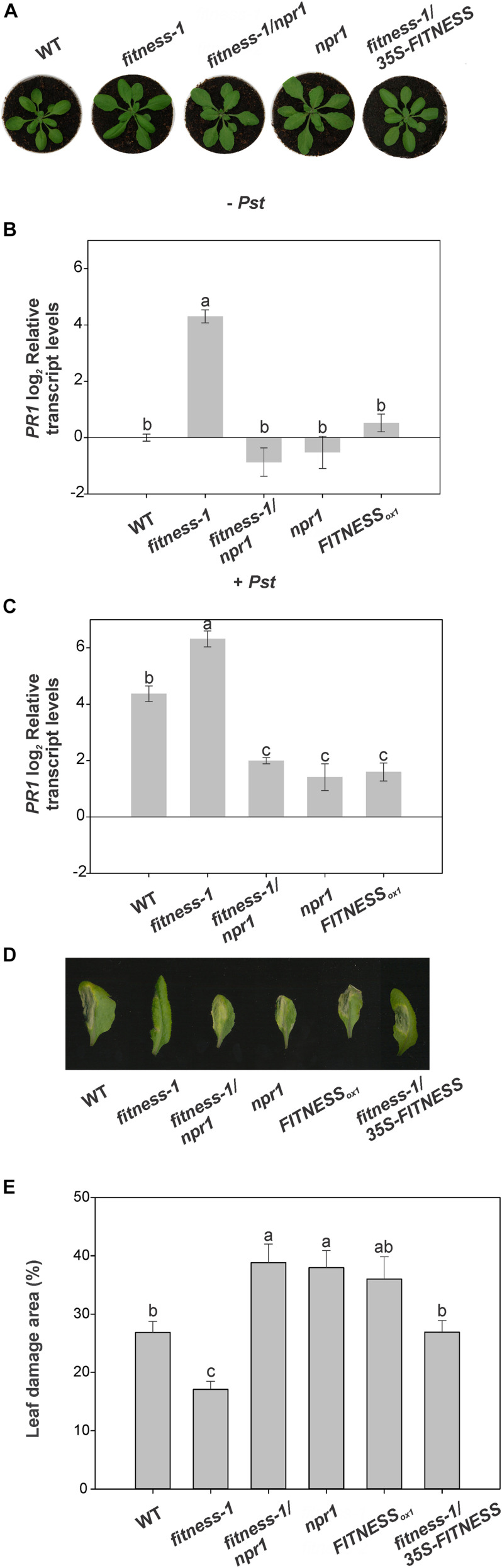
*Pst* resistance in *fitness* mutants is NPR1-dependent. **(A)** FITNESS requires NPR1 to induce *Pst* resistance in Arabidopsis. Representative photos of 25-day-old wild-type (WT), *fitness-1*, *fitness-1*/*npr1*, *npr1*, and *fitness-1*/*35S-FITNESS* plants. **(B,C)** Relative transcript levels of *PR1* in 25-day-old plants after 24 h mock treatment **(B)** or treatment with *Pst* **(C)**. The *fitness-1/npr1* mutant fails to induce *PR1*. Means of three biological replicates are shown. Error bars represent ± SE. **(D)** Disease symptoms of representative leaves of 4-week-old plants after *Pst* treatment. **(E)** Relative damage area of *Pst*-treated leaves. Samples were taken 6 days after infection. Values were averaged from at least nine biological replicates per genotype, and error bars represent SE. In panels **(B,C,E)**, bars with the same letter indicate no significant difference between samples (analysis of variance + Fisher least significant difference, *P* < 0.05).

The role of NPR1 as a central positive regulator of SAR transducing the SA signal to activate downstream *PR* gene expression has been well demonstrated, and mutations in *NPR1* lead to compromised disease resistance and loss of *PR* gene expression (Li et al., 2006). Several genes involved in the SA response are up-regulated in *fitness* mutants (Osella et al., 2018). To assess whether SA-triggered defense in *fitness* mutants is dependent on NPR1, we generated *fitness-1*/*npr1* double mutants by crossing the single mutants and screening for double homozygous mutants by a polymerase chain reaction in the F2 generation (Figure 2A and Supplementary Figure 1). We then tested the lines’ responses to *Pst* and measured *PR1* transcript levels as a proxy of SA response activation. As expected, WT plants showed a ∼20-fold elevation of *PR1* transcript abundance after *Pst* infection. Noteworthy, this level of *PR1* expression is similar to that observed in the *fitness-1* mutant before bacterial infection. Moreover, after infection, a further increase in *PR1* transcript level was observed in the *fitness-1* mutant, suggesting that this mutant can increase the defense responses beyond the defense levels observed in WT plants after infection. Results shown in Figure 2B indicate that a low level of *PR1* transcripts is present in the *fitness-1*/*npr1* double mutant, similar to that observed in *npr1*. However, after *Pst* infection, the major induction of *PR1* transcripts observed in the *fitness-1* mutant was absent in both the *npr1* and *fitness-1*/*npr1* mutants (Figure 2C). Altogether, these results suggest that the activation of the SA-signaling pathway in the *fitness* mutants requires active NPR1. Moreover, *fitness-1*/*npr1* mutants behave similarly to *npr1* mutants leading to high *Pst* counts after infection and to an increased size of the damaged area compared to those measured in WT plants and the *fitness-1* mutant reinforcing the fact that disease control in the *fitness* mutants is exerted through NPR1 signaling (Figures 2D,E).

### FITNESS Controls the Accumulation of Free and Conjugated SA

**FIGURE 3:**
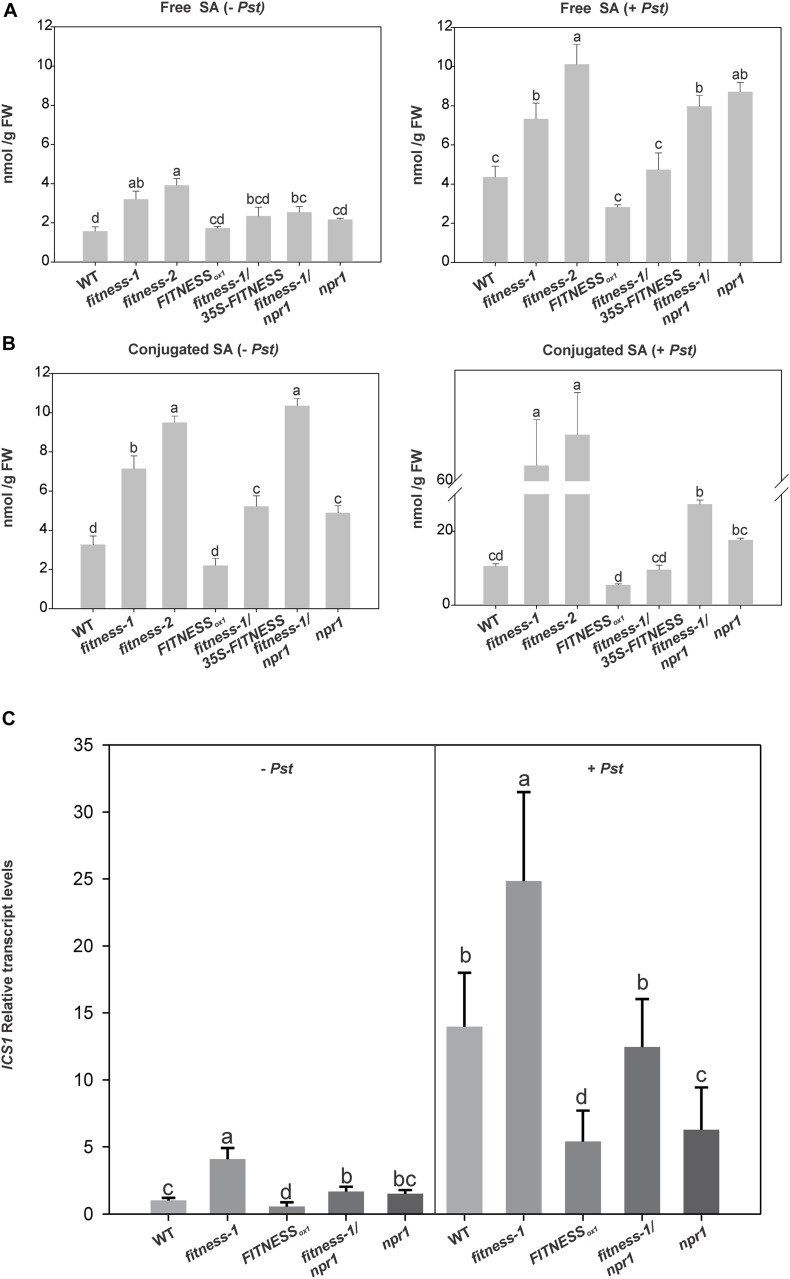
FITNESS controls the accumulation of free and conjugated SA. **(A,B)** Free and conjugated salicylic acid (SA) levels in plants with altered levels of *FITNESS*. Plants were mock treated (left panels) or *Pst* treated (right panels). Means of three biological replicates per genotype are shown. Error bars represent ± SE Bars with the same letter indicate no significant difference between samples (analysis of variance + Fisher least significant difference, *P* < 0.05). **(C)** Relative transcripts levels of *ICS1* in 25-day-old plants with altered level of *FITNESS* expression, mock-(left) or *Pst*-(right) infiltrated. Means of three biological replicates per genotype are shown. Error bars represent ± SE Bars with the same letter are not significantly different from one another (analysis of variance + Fisher least significant difference, *P* < 0.05).

As reported before, *fitness* mutants accumulate moderate levels of free SA relative to WT in control conditions (Osella et al., 2018). Thus, we quantitatively measured free and conjugated SA in plants with altered *FITNESS* levels in *Pst*-treated leaves after 24 h of infection and in mock-treated controls. In mock-infiltrated plants, besides the accumulation of free SA observed in *fitness* mutants, *FITNESS_ox__1_* plants had levels similar to WT plants (Figures 3A,B, left panels). Moreover, the *fitness-1*/*35S-FITNESS* line had lower free SA levels than the *fitness* mutants. An interesting feature was observed after *Pst* infection. All lines tested showed an increase in free SA compared to the control mock condition, indicating a further activation of SA synthesis (Figures 3A,B, right panels). Interestingly, *fitness-1*/*npr1* mutants showed SA levels similar to those in *fitness* mutants indicating that the failure in the double mutant’s defense responses is related to downstream signaling events. Even *FITNESS_ox__1_* plants showed an increase in free SA after *Pst* infection. However, this increase was less prominent than the one in WT plants. To explain all these observations, we measured the transcript levels of *ICS1*, which encodes for the enzyme ISOCHORISMATE SYNTHASE1 and is mainly responsible for the stress-induced accumulation of SA (Wildermouth et al., 2001). After mock infection, *ICS1* transcript levels in *fitness-1*/*npr1* mutants were similar to those measured in *npr1* mutants and lower than those measured in *fitness* mutants, reinforcing the idea that *FITNESS* modulates SA synthesis (Figure 3C). After *Pst* infection, *ICS1* transcript levels were additionally induced in all lines mirroring the measured SA levels (Figure 3C).

The levels of conjugated and free SAs showed similar trends, except for the *fitness-1*/*npr1* double mutant, which accumulated less conjugated SA than the *fitness* mutants after *Pst* infection (Figure 3B, right panel). Most of the SA is conjugated by SA-glucosyltransferases and transported to vacuoles for storage. Two genes in Arabidopsis encode active SA-glucosyltransferases (Thompson et al., 2017), *UDP-GLYCOSYLTRANSFERASE 74F1* (*UGT74F1*) and *UGT74F2*. We observed a significant increase in the transcript levels of *UGT74F2* in *fitness-1* mutant compared to WT (Osella et al., 2018).

Altogether, these results suggest that FITNESS acts as a negative regulator of SA biosynthesis. Enzymes, which influence flux and metabolite concentrations, are highly regulated. Knowing which genes affect certain metabolite levels open an opportunity to detect transcriptionally regulated pathways and novel genes controlling, or affecting, metabolic pathways (Redestig and Costa, 2011). The most commonly applied method to find metabolite-transcript co-responses is to calculate the Pearson correlation coefficient between the variables. To test the hypothesis that *FITNESS* and SA levels are linked, we calculated the Pearson correlation coefficient. We found a significant negative correlation between total SA content and *FITNESS* transcript levels (−0.65, *P* = 0.023), reinforcing the model that FITNESS is a new regulator of SA-mediated responses in Arabidopsis.

### Transcript Levels of Genes Involved in SA- and JA-Related Processes Are Altered in *FITNESS* Lines

As mentioned before, a genome-wide transcriptome study was previously done to identify genes present in the *FITNESS* transcriptomic network and one of the GO terms found significantly enriched was “response to SA” (SA, GO:0009751). Several genes related to SA-signaling like *ACCELERATED CELL DEATH 6* (*ACD6*), *LATE UPREGULATED IN RESPONSE TO HYALOPERONOSPORA PARASITICA* (*LURP1*), and *ICS1* showed increased expression in *fitness* mutants and a decreased expression in *FITNESS_ox__1_* plants. Also, as earlier stated, the transcripts of both *NPR1*, a key component of the SA signaling cascade, and *PR1*, a marker gene for SA signaling, were induced in *fitness* mutants (Osella et al., 2018). NPR1 is important for SA-induced SAR since *NPR1* loss-of-function plants are almost unable to activate *PR* genes’ expression. NPR1 was proposed to regulate *PR1* expression through its interaction with TGA TFs (Fan and Dong, 2002). In Arabidopsis, there are ten TGA TFs reported, and several interact constitutively with NPR1 (Zhou et al., 2000). However, two of them, namely TGA1 and TGA4, only interact with NPR1 upon SA induction (Després et al., 2003). Interestingly, only these two TFs showed increased transcript levels in *fitness* mutants (Osella et al., 2018); of note, TGA1 is one of the proteins that interact with FITNESS in a yeast two-hybrid assay (Trigg et al., 2017).

**FIGURE 4:**
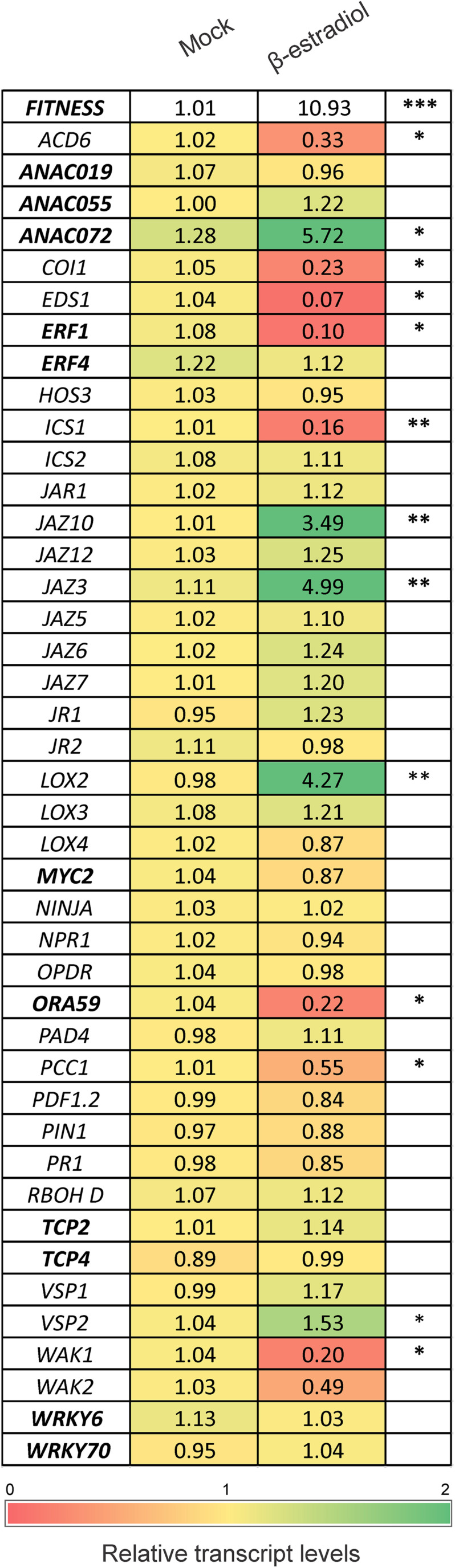
Relative transcript levels of defense-/stress-related genes possibly involved in the *FITNESS* regulatory network. Expression of selected genes possibly involved in the *FITNESS* regulatory network. Heat map showing differential expression of genes in the *FITNESS*-IOE line after 4 h of treatment with 10 μM estradiol vs. mock treatment. Data are expressed as relative transcript levels accompanied with Student’s *t*-test (*P < 0.05; ** *P* < 0.01; *** *P* < 0.001). Values were averaged from at least three biological replicates per treatment. Green and red colors denote up- and down-regulated genes, respectively, compared to mock treatment. Genes encoding for TFs are highlighted in bold.

To further characterize the FITNESS regulatory network, we used estradiol (EST) – inducible overexpressing (*FITNESS*-IOE) plants. We first tested EST-dependent *FITNESS* expression 2, 4, and 6 h after EST treatment, using 15-day-old *FITNESS*-IOE plants. As controls, we used 0.01% Silwet 77-treated *FITNESS*-IOE lines. *FITNESS* expression increased by more than 10-fold after 4 h EST treatment, reaching its maximum. Six hours after the treatment, *FITNESS* transcript levels declined again (Supplementary Figure 3). To find possible FITNESS target genes, we used 15-day-old *FITNESS*-IOE seedlings harvested 4 h after either treatment with 0.01% Silwet 77 (control) or 10 μM EST and performed a targeted transcript profiling. We selected 42 genes and checked their expression by qPCR. The list included 31 non-TF- and 11 TF-encoding genes that were either significantly altered in *fitness* mutants or involved in specific hormone or stress signaling pathways (Figure 4). Among them, four genes (*LOX2*, *ANAC072*, *JAZ3*, and *JAZ10*) were highly induced, and eight were significantly repressed (*ICS1*, *COI1*, *WAK1*, *PCC1*, *ACD6*, *EDS1*, *ORA59*, *ERF1*) 4 h after the induction of *FITNESS* expression (Figure 4).

Noteworthy, many of these putative target genes are involved in JA synthesis or signaling. *LOX2* (*LIPOXYGENASE2*) encodes a key enzyme in the octadecanoid pathway leading to JA biosynthesis (Bell et al., 1995). *ANAC072*, together with the homologs *ANAC019* and *ANAC055*, act in the JA-mediated inhibition of the SA pathway by suppressing *ICS1* expression. Accordingly, the transcripts levels of *ICS1* were significantly repressed in *FITNESS*-IOE lines, which have higher transcript levels of *ANAC072* (Figure 4). Also, *JAZ3* and *JAZ10*, which act by inhibiting JA-responsive genes, were induced in *FITNESS*-IOE lines. However, *COI1* (*CORONATINE INSENSITIVE 1*) transcript levels were significantly reduced. *COI1* encodes an F-box protein of the Skp/Cullin/F-box complex (SCF^*COI*1^). It is an essential component of the bioactive JA perception apparatus, and is required for most JA-signaling processes (Katsir et al., 2008). It has been reported that Methyl Jasmonate (MeJA) inhibits root growth in Arabidopsis (Staswick et al., 1992). Therefore, we examined whether primary root growth in Arabidopsis plants with altered expression levels of *FITNESS* was sensitive to MeJA. *FITNESS_ox__1_* line roots were significantly less inhibited than WT roots (43 vs. 52%) in the presence of 50 μM MeJA when compared to control conditions. On the other side, *fitness* mutants showed the highest growth inhibition (62 and 66% for *fitness-1* and *-2*, respectively). Similar trends were observed when the roots were grown in the presence of 10 μM MeJA (Supplementary Figure 4). These results suggest that FITNESS modulates JA-related responses.

We also analyzed the JA signaling branches downstream of JAZ repressors, namely, the MYC TF branch, which is related to wound responses (with *VEGETATIVE STORAGE PROTEIN2* [*VSP2*] as a marker gene), and the ERF TF branch, which links JA to necrotrophic pathogen resistance (with *PLANT DEFENSIN1.2* [*PDF1.2*] as a marker gene) in *FITNESS*-IOE lines at 6h after estradiol induction. *VSP1* and *2* transcript levels showed a significant induction (Supplementary Figure 5). On the other side, expression of *PDF1.2*, which is a marker gene for JA-ethylene (ET)-mediated responses, was significantly decreased 6h after *FITNESS* induction. This is in agreement with the fact that *PDF1.2* is a direct target of the ERF TFs ORA59 and ERF1, and expression of the genes encoding them was strongly reduced in *FITNESS*-IOE lines.

### Reproductive Output of *Fitness* Mutants After *Pst* Treatment

**FIGURE 5:**
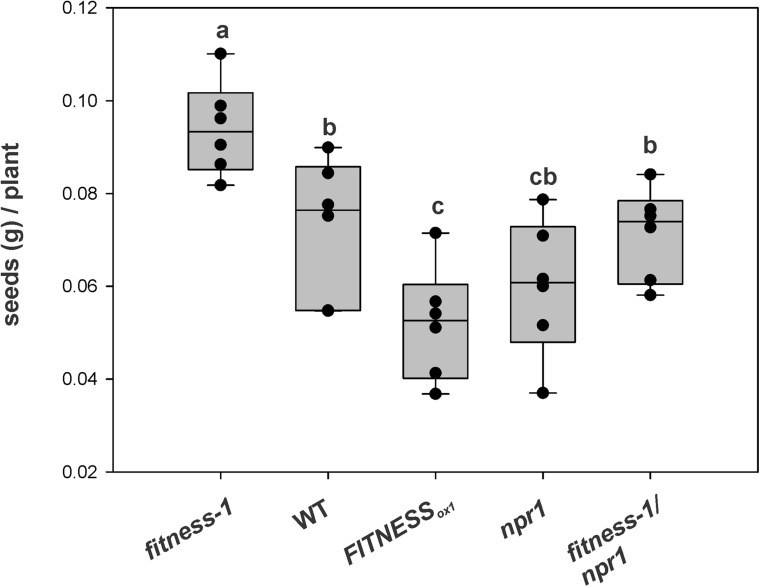
Seed productivity of different *FITNESS* lines after pathogen attack. At least six plants per line were sprayed with a *Pst* suspension at approximately 10^8^ c.f.u. ml^–1^. Bars with the same letter indicate no significant difference between samples (analysis of variance + Fisher least significant difference, *P* < 0.05). The experiment was repeated twice with similar results.

Based on the concept that an active immune system is associated with production costs, we aimed to measure the reproductive output of *fitness* mutants after *Pst* treatment. To this end, the different lines and mutants were sprayed with a *Pst* suspension at approximately 10^8^ c.f.u. ml^–1^, and then were allowed to grow until seed set. The quantification showed that the total seed yield per plant was higher in the *fitness-1* mutant than all other lines (Figure 5). The seed yield of the *fitness-1*/*npr1* double mutant was similar to that of WT plants leading to the model that in *fitness* mutants, the higher yield was related to their elevated SA content (see above). This observation opens up new possibilities for the generation of plants combining resistance traits without compromising yield.

## Discussion

In this study, the robust resistance of *fitness* mutants, and the enhanced sensitivity upon *FITNESS* overexpression, demonstrate the importance of FITNESS as a negative regulator of plant immunity in response to infection by the hemibiotrophic pathogen *Pst*. The induction or repression of defense genes is orchestrated by signaling networks directed by plant hormones, of which SA and JA are the major players (Caarls et al., 2015). Moreover, NPR1 acts as a master key in plant defense signaling networks mediating cross-talk between SA and JA/ET responses (Backer et al., 2019). Global gene expression studies revealed that FITNESS functions to modulate SA-responsive gene expression. Also, the levels of free and conjugated SA were negatively linked to *FITNESS* expression. *FITNESS* loss of function leads to the accumulation of both SA forms. On the contrary, *FITNESS* overexpression reduces the phytohormone synthesis and accumulation. We further confirmed that the *fitness-1*/*35S-FITNESS* line accumulates lower SA levels than the *fitness* mutants supporting the model that FITNESS negatively influences SA synthesis. We also showed that the basal SA content in *fitness* mutants provides the benefit of limiting bacterial pathogen infection. However, SA accumulation after *Pst* infection in *fitness* mutants does not depend on the presence of active NPR1. This conclusion can be drawn from the fact that both the *fitness-1*/*npr1* double mutant and the *fitness-1* single-gene mutant accumulate similar SA levels after *Pst* infection. This agrees with previous reports that show NPR1 is not necessary for SA synthesis (Marek et al., 2010). However, the level of conjugated SA, which accounts for its storage form, is decreased in the *fitness-1*/*npr1* double mutant. Plants unable to synthesize or accumulate SA are more susceptible to infection by certain pathogens (van Wees and Glazebrook, 2003). This is consistent with the increased susceptibility to *Pst* infection in the *fitness-1*/*npr1* compared to the *fitness-1* mutant. As mentioned before, SA-glucosyltransferases convert SA to its conjugated form. We found *UGT74F2* to be transcriptionally induced in the *fitness-1* mutant, in agreement with high conjugated SA levels after *Pst* infection. By searching in publicly available transcriptome data, we also observed that *UGT74F1* and *UGT74F2* are transcriptionally down-regulated in the *npr1* mutant compared to WT after SA treatment^5^ (GSE51626; Singh et al., 2015). Taking all this into account, we conclude that NPR1 is essential in *fitness* mutants for SA storage and defense activation but not for SA synthesis after *Pst* infection.

In Arabidopsis, simultaneous activation of SA and JA mediated resistance is restricted by the strong negative effect of SA on the JA/ET responses (Spoel and Dong, 2008; Pieterse et al., 2009). Notably, altered levels of *FITNESS* led to alteration of the network of TFs, which control defense responses. After *FITNESS* inducible expression, *ANAC072* transcript levels were significantly induced, while *ORA59* and *ERF1* transcript levels were significantly repressed. Recent studies showed that ANAC072 and its homologs, ANAC019 and ANAC055, are involved in plants’ response to bacterial pathogens, JA-mediated defense, and thermotolerance (Bu et al., 2008; Zheng et al., 2012; Guan et al., 2014). Also, ANAC072 acts by inhibiting *ICS1* expression (Zheng et al., 2012) and accordingly, *ICS1* transcript levels were downregulated in *FITNESS*-IOE plants.

*ORA59* and *ERF1*, which integrate JA and ET signals to promote antimicrobial compounds’ expression, were strongly downregulated in *FITNESS*-IOE lines. Also, the expression of *COI1* was strongly downregulated in *FITNESS*-IOE lines. Considering that *COI1* loss of function abolishes JA-dependent responses and that *ORA59* functions downstream of *COI1*, we propose that elevated *FITNESS* expression leads to an inhibition of JA responses. We also found that transcripts of two JAZ proteins, namely JAZ3 and JAZ10, which act as repressors of JA signaling, are highly upregulated after *FITNESS* induction. Arabidopsis possesses thirteen *JAZ* genes, and the specific role of each one is still unclear. Proteasomal degradation of JAZ proteins results in JA responses’ derepression and activation of JA-responsive genes (Memelink, 2009). The aforementioned results suggest that JAZ degradation upon the perception of biologically active JAs, and JA perception itself, are affected due to *COI1* repression.

Summarizing, our work demonstrates that in *fitness* mutants, changes induced in the transcriptional network lead to an efficient defense response without a compromise in plant productivity after bacterial pathogen attack. Although this is in conflict with the proposed incompatibility between growth and defense, growth inhibition due to activation of defense responses is not a default program. For example, Arabidopsis accession C24 has no yield penalties, although it has an elevated pathogen resistance due to constitutive high levels of disease resistance (Bechtold et al., 2010). Additionally, by analyzing the reported seed yield of several mutants known for their differential SA accumulation, no predictive pattern can be envisaged between yield and defenses activation. *cpr6-1* and *lsd1* mutants both accumulate SA and show increased defense responses but low seed yields (Dietrich et al., 1997; Clarke et al., 1998; Mateo et al., 2004; Bechtold et al., 2010; Bernacki et al., 2019) and *acd5* mutants which also accumulate SA, have decreased seed yield and decreased tolerance to pathogens (Greenberg et al., 2000). On the contrary, higher seed yields were reported in SA accumulation affected lines (van Wees and Glazebrook, 2003; Abreu and Munné-Bosch, 2009; Lu et al., 2009). The impact of FITNESS on reproductive performance was previously reported for plants grown under non-stress conditions (Osella et al., 2018). Here, we go beyond our initial observation and show that a lack of FITNESS leads to reproductive success even after a bacterial pathogen attack. Plant diseases are a constant threat to agricultural production. Yield losses due to pathogen attacks include direct and indirect consequences. On one side, the yield might be compromised, but also crop pests are critical concerning food security. A significant proportion of global crop production is annually lost due to pests and diseases (Savary et al., 2012). While pesticides help reduce these losses, there is growing concern about pesticide resistance and their impacts on health and the environment (Schwarzenbacher et al., 2014). Therefore, the identification and downregulation of *FITNESS* orthologs opens new avenues for future research in improving crop species toward reducing the impact of pests and diseases on crop yield to support global food production. In conclusion, we presented evidence that FITNESS is a new component of the plant defense response network and an integration node for balancing the reproductive output during stress responses.

## Data Availability Statement

The original contributions presented in the study are included in the article/Supplementary Material, further inquiries can be directed to the corresponding author.

## Author Contributions

DM performed most of the experiments. LRT isolated and characterized the *fitness-1*/*npr1* double mutant. DM and SB designed and constructed the *FITNESS* inducible overexpressing line. MIZ conceived the project and wrote the manuscript with contributions by all authors.

## Conflict of Interest

The authors declare that the research was conducted in the absence of any commercial or financial relationships that could be construed as a potential conflict of interest.

## Footnotes

1 http://www.ncbi.nlm.nih.gov/

2 http://www.arabidopsis.org/

3 http://www.genscript.com/wolf-psort.html

4 http://sumosp.biocuckoo.org/online.php

5 https://www.ncbi.nlm.nih.gov/gds
