# Supplementary figures and images for: FITNESS Acts as a Negative Regulator of Immunity and Influences the Plant Reproductive Output After Pseudomonas syringae Infection
Source: Front Plant Sci. 2021 Feb 4;12:606791. doi: 10.3389/fpls.2021.606791 (PMC7889524; doi:10.3389/fpls.2021.606791)

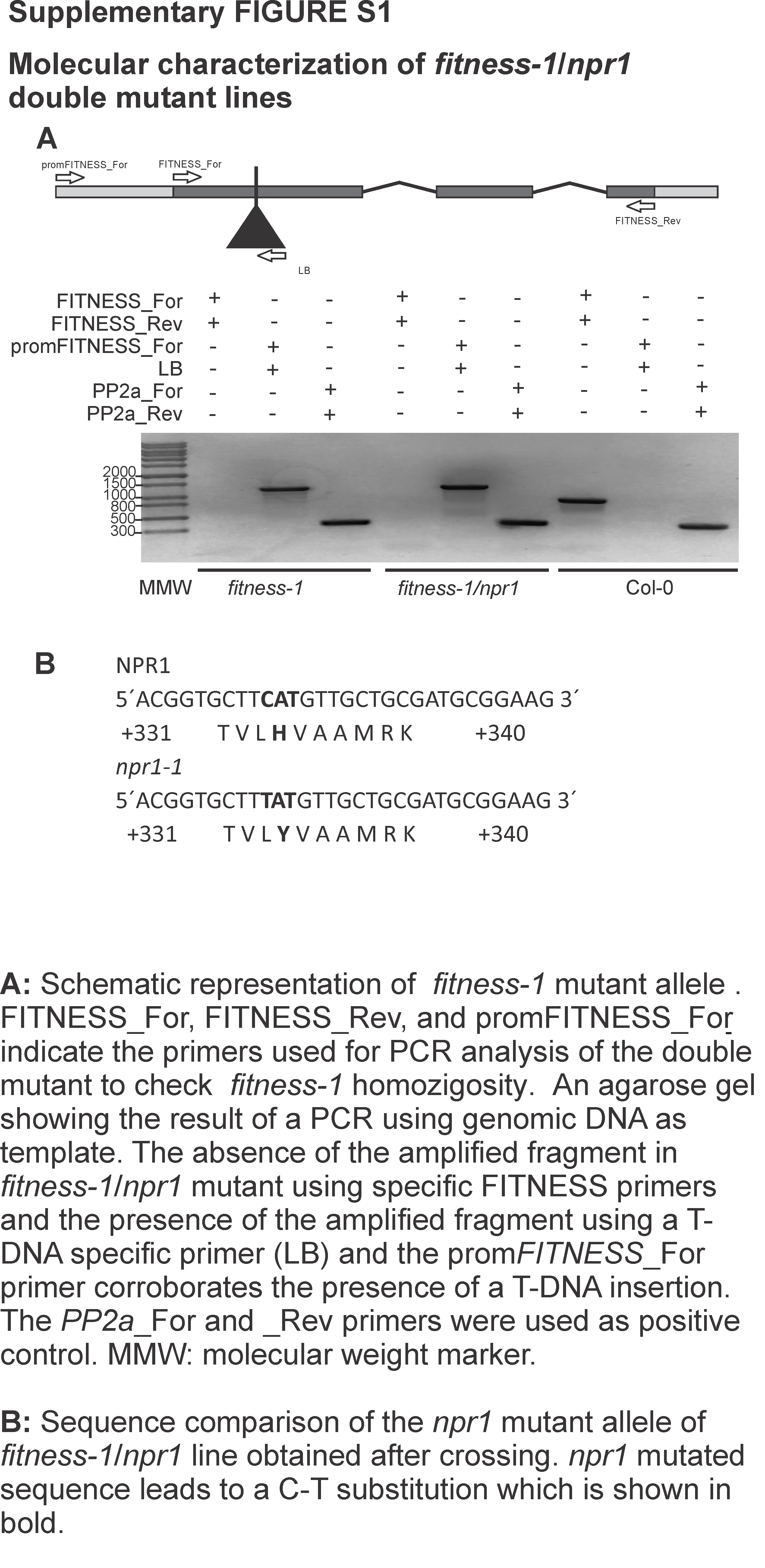

Supplement: Supplementary file 1 [file Image_1.TIF]

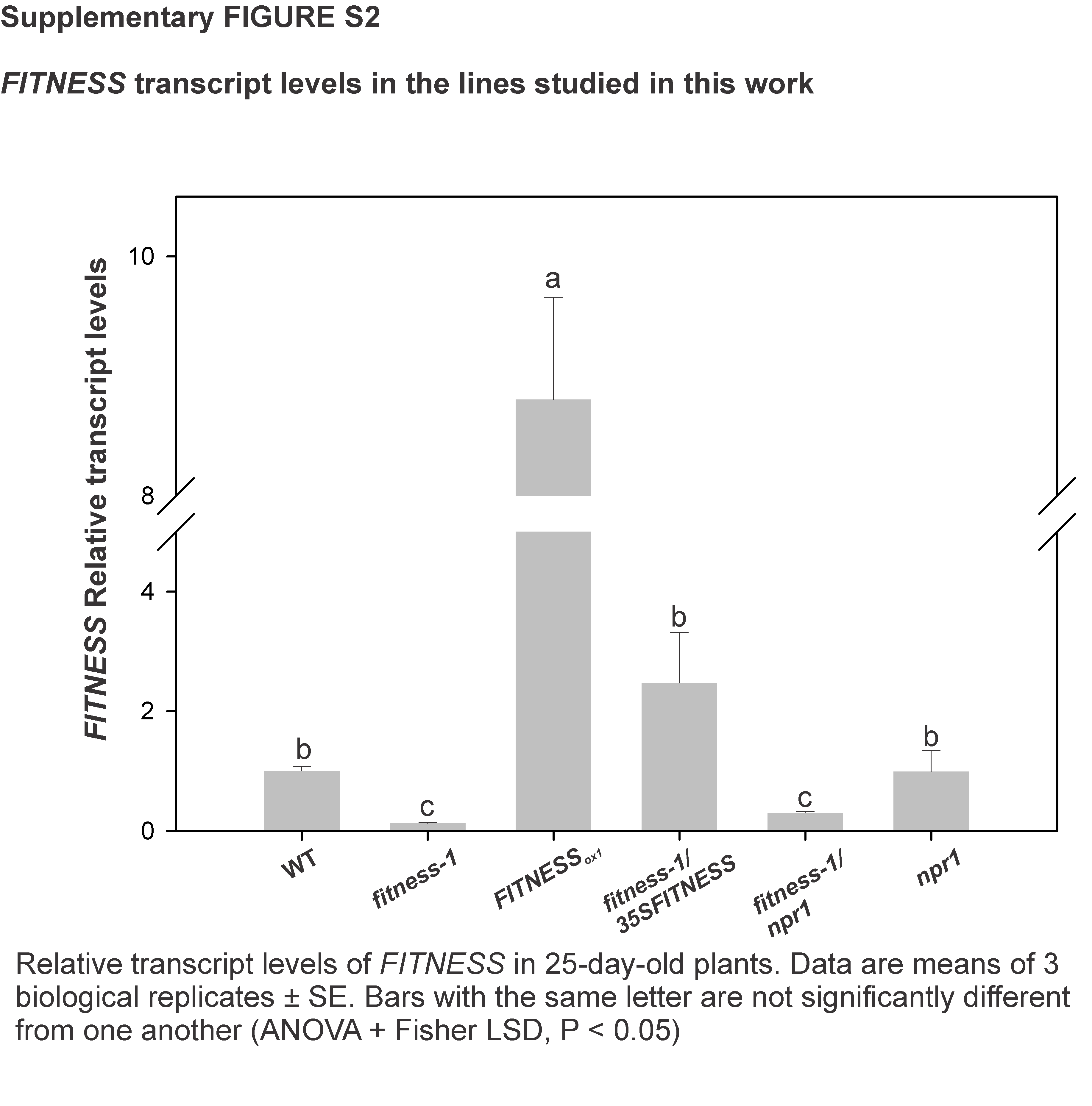

Supplement: Supplementary file 2 [file Image_2.TIF]

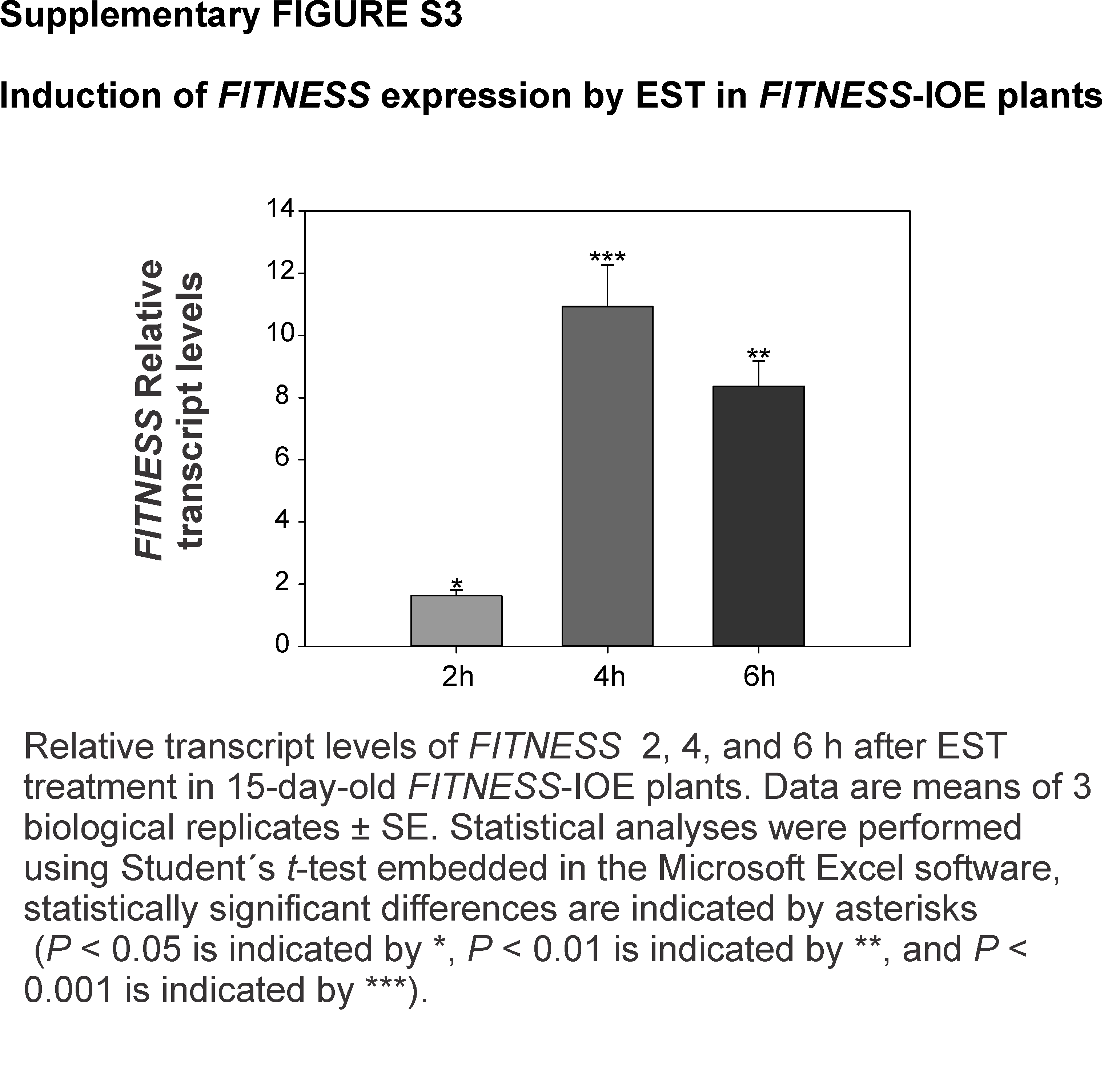

Supplement: Supplementary file 3 [file Image_3.TIF]

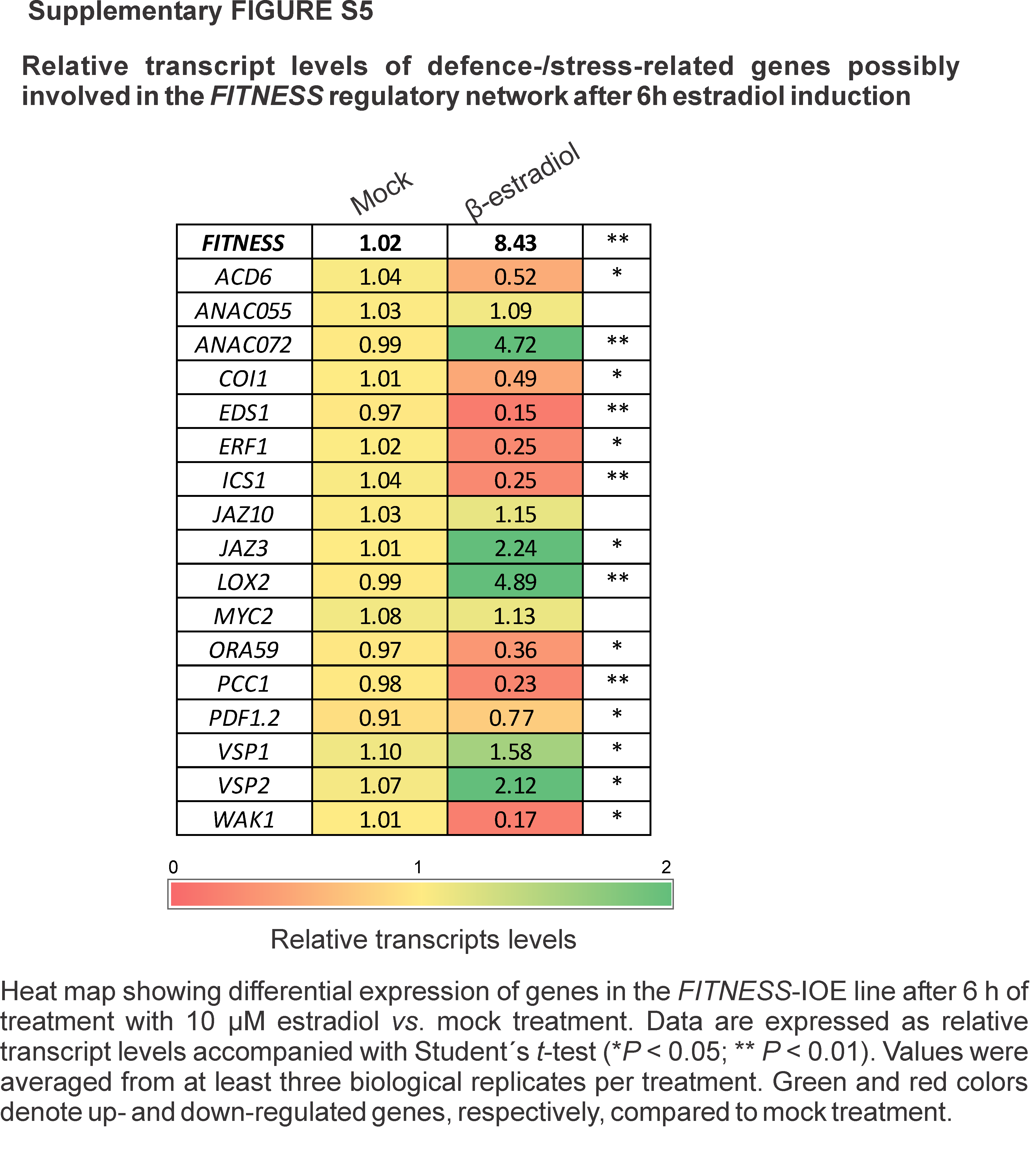

Supplement: Supplementary file 4 [file Image_4.TIF]
