# Supplementary material for: FITNESS Acts as a Negative Regulator of Immunity and Influences the Plant Reproductive Output After Pseudomonas syringae Infection
Source: Front Plant Sci. 2021 Feb 4;12:606791. doi: 10.3389/fpls.2021.606791 (PMC7889524; doi:10.3389/fpls.2021.606791)
Supplement: Supplementary file 6 [file Table_1.DOC]

**Supplementary Table S1**

Sequences of the primers used in this study

**PRIMERS USED FOR CLONING AND PCR mutant confirmation**

| **Name** | | **Sequence** | |
| --- | --- | --- | --- |
| FITNESS_For | 5´GGATCCATGGATACACAAAGGTTGCC3´ | | |
| FITNESS*_*Rev | 5´TCTAGATTAAGTCTCTCTTTTCACGAAC3´ | | |
| LB | 5´GCGTGGACCGCTTGCTGCAACT3´ | | |
| promFITNESS_For | 5´TCTAGAAATTCTGAAGAAGAACAGGG | | |
| npr1_*_*For | 5´TCTTGCCGATGTCAACCATA3´ | | |
| npr1*_*Rev | 5´ATGAGTGCGGTTCTACCTTC3´ | | |
| FITNESS-IOE *Pac*I | 5´ ttaattaATGGATACACAAAGGTTGCC3´ | | |
| FITNESS-IOE *Spe*I | 5´ actagTTAAGTCTCTCTTTTCACGAAC3´ | | |
|  |  | | |
| **PRIMERS USED FOR qPCR** | | |  |
| **Name** | | **Sequence** | |
| *PP2a_For_At1g13320* | | 5´CCTGCGGTAATAACTGCATCT3´ | |
| *PP2a_Rev_At1g13320* | | 5´CTTCACTTAGCTCCACCAAGCA3´ | |
| *FITNESS_At1g07050_For* | | 5´ACAAAGACTTGGGTCTGAAGCTG3´ | |
| *FITNESS_At1g07050_Rev* | | 5´TCTGGTGATCAGACCAAGCATCG3´ | |
| *ICS1_At1g74710_For* | | 5´GCTTGGCTAGCACAGTTACAGC3´ | |
| *ICS1_At1g74710_Rev* | | 5´CACTGCAGACACCTAATTGAGTCC3´ | |
| *ICS2_At1g18870_For* | | 5´GCCTAGAGGTGATTCAAAGGTTCG3´ | |
| *ICS2_At1g18870_Rev* | | 5´ACTCAAGATCGTCTTTGGGACTGG3´ | |
| *NPR1_At1g64280_For* | | 5´AACGATTCTTCCCGCGCTGTTC3´ | |
| *NPR1_At1g64280_Rev* | | 5´TTCTCCGCAAGCCAGTTGAGTC3´ | |
| *PR1_At2g14610_For* | | 5´ACATGTGGGTTAGCGAGAAGGC3´ | |
| *PR1_At2g14610_Rev* | | 5´AAACTCCATTGCACGTGTTCGC3´ | |
| *NINJA_AT4G28910_For* | | 5´AGTTCCATCAAACTCAGCTACTGC3´ | |
| *NINJA_AT4G28910_Rev* | | 5´TGTTGTCTCCCGTTGGTCGTTC3´ | |
| *JAZ3_AT3G17860_For* | | 5´CTGCCCAGTACCAGTTTCTTGTTC3´ | |
| *JAZ3_AT3G17860_Rev* | | 5´AGCCAGAGGTAATGCCACTGTTG3´ | |
| *JAZ5_AT1G17380_For* | | 5´TTCCAAAGGCGAACCCTCTACC3´ | |
| *JAZ5_AT1G17380_Rev* | | 5´TCCTGGCTGTGATTCACTGAGG3´ | |
| *JAZ6_AT1G72450_For* | | 5´TTCCCGATCTTAACGAGCCAACG3´ | |
| *JAZ6-AT1G72450_Rev* | | 5´ACCTGATGTTGCTGCCCAGTTT3´ | |
| *JAZ7_AT2G34600_For* | | 5´TGCGACTTGGAACTTCGCCTTC3´ | |
| *JAZ7_AT2G34600_Rev* | | 5´AGCTGCTTGATTCGTCCAACGAG3´ | |
| *JAZ10_AT5G13220_For* | | 5´CAAACCAACAACGCTCCTAAGCC3´ | |
| *JAZ10_AT5G13220_Rev* | | 5´TCGCACCTTGAATATCTCGGAAAC3´ | |
| *JAZ12_AT5G20900_For* | | 5´TGCACAGCCATTTCCTATTCAGC3´ | |
| *JAZ12_AT5G20900_Rev* | | 5´GCCTCCTTGCAATAGGTAGATCAG3´ | |
| *COI1_AT2G39940_For* | | 5´TGCACTTCCGACGGATGATTGTC3´ | |
| *COI1_AT2G39940_Rev* | | 5´TCCTGCAGTGTGTAACGATGCTC3´ | |
| *JAR1_AT2G46370_For* | | 5´AGCAACTAGCGCAGGTACTCTC3´ | |
| *JAR1_AT2G46370_Rev* | | 5´AGTTTCAACCTTCTCCAACATCCC3´ | |
| *ORA59_AT1G06160_For* | | 5´AGGGATAAGAGTGTGGCTTGGG3´ | |
| *ORA59_AT1G06160_Rev* | | 5´CGAAAGCCGCCTGATCATAAGC3´ | |
| *ANAC019_AT1G52890_For* | | 5´TCTAACCCAAACCGCATCTCGTC3´ | |
| *ANAC019_AT1G52890_Rev* | | 5´AGTATCCGCAAGACCGTGGAAC3´ | |
| *ANAC055_AT3G15500_For* | | 5´CGTCGAAATGGAAGCACCAAGC3´ | |
| *ANAC055_AT3G15500_Rev* | | 5´TGTCGACGAACCATTGTTGCTG3´ | |
| *ANAC072_AT4G27410_For* | | 5´ATTATCACGGCGGATGGTCGTC3´ | |
| *ANAC072_AT4G27410_Rev* | | 5´ACTTGGAGCTTCCATGGCTACG3´ | |
| *MYC2_AT1G32640_For* | | 5´AGCCACTAAACCACGTCGAAGC3´ | |
| *MYC2_AT1G32640_Rev* | | 5´TACAACCGCTCGTAACGCGTAG3´ | |
| *ERF1_AT3G23240_For* | | 5´TCCCTTCAACGAGAACGACTCAG3´ | |
| *ERF1_AT3G23240_Rev* | | 5´AGGTTTGTTGCGTGGACTGCTC3´ | |
| *JR2_AT4G23600_For* | | 5´CGAAGCCTGCACCTTCTTATGGAC3´ | |
| *JR2_AT4G23600_Rev* | | 5´TCGTCTTCGATGTCCACAAAGCTC3´ | |
| *JR1_AT3G16470_For* | | 5´CCTGTCCTTGGAAGTGATCATGGG3´ | |
| *JR1_AT3G16470_Rev* | | 5´TCATCTGGTCCAAGCACAAACTCC3´ | |
| *OPDR_AT1G09400_For* | | 5´GTCGCGTTTCTCATCAAGATTGTC3´ | |
| *OPDR*_*AT1G09400_Rev* | | 5´TCGGCGAATGGCTTATCTGTTG3´ | |
| *LOX2_AT3G45140_For* | | 5´CGAGAGGTCTCGATGACATTGCTG3´ | |
| *LOX2_AT3G45140_Rev* | | 5´TGCGTAGTCTTCTACCGTAATCCG3´ | |
| *LOX3_AT3G22400_For* | | 5´CCCTTGCCTGACATCCTCAAAGAG3´ | |
| *LOX3_AT3G22400_Rev* | | 5´CTCGAGCAAACTCTTCGTCAGTCC3´ | |
| *LOX4_At1G72520_For* | | 5´GGAAGACCACATCATCGGTCAAC3´ | |
| *LOX4_At1G72520_Rev* | | 5´AAACGGTTCGTCTCTAACGCTTG3´ | |
| *EDS1_At3g48090_For* | | 5´GCTCAATGACCTTGGAGTGAGC3´ | |
| *EDS1_At3g48090_Rev* | | 5´TCTTCCTCTAATGCAGCTTGAACG3´ | |
| *PAD4_At3g52430_For* | | 5´AGATACGCGAGCACAACGCAAG3´ | |
| *PAD4_At3g52430_Rev* | | 5´TTCTCGCCTCATCCAACCACTC3´ | |
| *ACD6_At4g14400_For* | | 5´TCTGCCACTTGGTTTGCGATGG3´ | |
| *ACD6_At4g14400_Rev* | | 5´ACTCCTCCGCTGTGAGAGAAATC3´ | |
| *ERF4_AT3G15210_For* | | 5´CTGTGACATCGGCGTTTAGATCG | |
| *ERF4_AT3G15210_Rev* | | 5´TTGGGCACCACAAGCCATCTT C | |
| *HOS3_AT4G36830_For* | | 5´TGGTTTACCCGGTTCGGCTTTC3´ | |
| *HOS3*_*AT4G36830_Rev* | | 5´ACACGAGATTACAGCCCACGAG3´ | |
| *PCC1*_*At3g22231_For* | | 5´TGTATCTTCTGCTGCGGCGTATG3´ | |
| *PCC1*_*At3g22231_Rev* | | 5´TTGGGCAACGACTTCTGTCTCATC3´ | |
| *PDF1.2*_*At5g44420_For* | | 5´CCATCATCACCCTTATCTTCGC3´ | |
| *PDF1.2*_*At5g44420_Rev* | | 5´TGTCCCACTTGGCTTCTCG3´ | |
| *PIN1_AT1G73590_For* | | 5´GGCATGGCTATGTTCAGTCTTGGG3´ | |
| *PIN1_AT1G73590_Rev* | | 5´GCTGCTCTTCTGTTTCCACAAGC3´ | |
| *RboH D*_*AT5G47910_For* | | 5´ATGATCAAGGTGGCTGTTTACCC3´ | |
| *RboH D*_*AT5G47910_Rev* | | 5´ATCCTTGTGGCTTCGTCATGTG3´ | |
| *TCP2_AT4G18390_For* | | 5´CTCAAGCTCCTCTGTTTCTTCTGC3´ | |
| *TCP2*_*AT4G18390_Rev* | | 5´TTGTCTGCAGCTGCTGTTGTTTG3´ | |
| *TCP4*_*AT3G15030_For* | | 5´GTTTCAGGGCTTGTCTAGCTTCC3´ | |
| *TCP4*_*T3G15030_Rev* | | 5´ACCGAGAACACCTTCCCTGTTC3´ | |
| *VSP1*_*At5g24780 For* | | 5´TCGAGAATCTCAAGGCTGTTGGTG3´ | |
| *VSP1*_*At5g24780_Rev* | | 5´TCAACTTCGATCCGTTTGGCTTG3´ | |
| *VSP2*_*At5g24770_For* | | 5´GTTAGGGACCGGAGCATCAA3´ | |
| *VSP2*_*At5g24770_Rev* | | 5´AACGGTCACTGAGTATGATGGGT3´ | |
| *WAK1*_*AT1G21250_For* | | 5´ACCTTTCAGCTGGTTGCCAAGAC3´ | |
| *WAK1*_*AT1G21250_Rev* | | 5´AGCCTCCAACCTTGTTTCTACAGG3´ | |
| *WAK2*_*AT1G21270_For* | | 5´TGCCCATCTGGTTACCGCAAAG3´ | |
| *WAK2*_*AT1G21270_Rev* | | 5´AGAAGCCGATGGTGGTTCCAAG3´ | |
| *WRKY6*_*AT1G62300_For* | | 5´TGTCCCGTTCGCAAACAAGTTC3´ | |
| *WRKY6*_*AT1G62300_Rev* | | 5´GGTTATGGTTTCCCTCGTAGGTTG3´ | |
| *WRKY70*_*At3g56400_For* | | 5´TGAGCTCGAACCCAAGATGTTCAG3´ | |
| *WRKY70*_*At3g56400_Rev* | | 5´TGCTCTTGGGAGTTTCTGCGTTG3´ | |
